# Impact of Different Titanium Implant Thread Designs on Bone Healing: A Biomechanical and Histometric Study with an Animal Model

**DOI:** 10.3390/jcm8060777

**Published:** 2019-05-31

**Authors:** Sergio Alexandre Gehrke, Tiago Luis Eliers Treichel, Letícia Pérez-Díaz, Jose Luis Calvo-Guirado, Jaime Aramburú Júnior, Patricia Mazón, Piedad N. de Aza

**Affiliations:** 1 Department of Research, Biotecnos CP 11100-Montevideo, Uruguay; jaimearamburujunior@gmail.com; 2 Instituto de Bioingenieria, Universidad Miguel Hernández, Avda. Ferrocarril s/n, 03202 Elche (Alicante), Spain; piedad@umh.es; 3 Department of Anatomy, Faculty of Veterinary, Universidade de Rio Verde, 104, Rio Verde-GO 75901-970, Brazil; tiago@unirv.edu.br; 4 Laboratorio de Interacciones Molecular, Facultad de Ciencias, Universidad de la Republica, Calle Iguá 4225, 11400 Montevideo, Uruguay; letperez@gmail.com; 5 Department of Oral and Implant Surgery, Faculty of Health Sciences, Universidad Católica de Murcia (UCAM), 30107 Murcia, Spain; jlcalvo@ucam.edu; 6 Departamento de Materiales, Óptica y Tecnologia Electrónica, Universidad Miguel Hernández, Avda. Universidad s/n, 03202 Elche (Alicante), Spain; pmazon@umh.es

**Keywords:** animal study, dental implants, implant design, healing chamber, thread design

## Abstract

Threads of dental implants with healing chamber configurations have become a target to improve osseointegration. This biomechanical and histometric study aimed to evaluate the influence of implant healing chamber configurations on the torque removal value (RTv), percentage of bone-to-implant contact (BIC%), bone fraction occupancy inside the thread area (BAFO%), and bone and osteocyte density (Ost) in the rabbit tibia after two months of healing. Titanium implants with three different thread configurations were evaluated: Group 1 (G1), with a conventional “v” thread-shaped implant design; Group 2 (G2), with square threads; and Group 3 (G3), the experimental group with longer threads (healing chamber). Ten rabbits (4.5 ± 0.5 kg) received three implants in each tibia (one per group), distributed in a randomized manner. After a period of two months, the tibia blocks (implants and the surrounding tissue) were removed and processed for ground sectioning to evaluate BIC%, BAFO%, and osteocyte density. The ANOVA one-way statistical test was used followed by the Bonferoni’s multiple comparison test to determine individual difference among groups, considering a statistical difference when *p* < 0.05. Histometric evaluation showed a higher BAFO% values and Ost density for G3 in comparison with the other two groups (G1 and G2), with *p* < 0.05. However, the RTv and BIC% parameters were not significantly different between groups (*p* > 0.05). The histological data suggest that the healing chambers in the implant macrogeometry can improve the bone reaction in comparison with the conventional thread design.

## 1. Introduction

Long-term investigations have documented the high predictability of implant-supported restorations for fully and partially edentulous patients [1,2]. However, the survival of implant-supported restorations placed in bone with low density (posterior maxilla) present inferior rates when compared to dental implants placed in areas with higher bone density, as in the anterior mandible [3,4]. The demand for improved predictability of dental implants in sites with lower bone density has led researchers and industry to develop new implant designs to improve response in these areas. In this sense, different surface treatments models [5,6] and different macrogeometric designs were developed [7,8].

The initial implant stability is a fundamental requirement to obtain osseointegration. Thus, the selection of an implant that will provide adequate stability in bone of poor quality is important. A conical implant macrogeometry can provide adequate stability because it creates pressure on cortical bone in areas of reduced bone quality [9]. Preclinical animal and clinical human studies have showed that the conical implant design can affect the primary stability and the osseointegration events [10,11]. In addition to the shape of the implant body, the thread design should provide for improved stability and implant to bone contact. An ideal implant scheme should provide a balance between compressive and tensile forces while minimizing shear force generation during the installation [12].

Previous animal studies [13,14] have demonstrated that alterations in the proportion between the osteotomy and the implant diameter to promote spaces filling with blood (healing chambers) could improve the osseointegration process. Initially, the surgical technique to install the implants advocated a close fit between the bone and implant after the osteotomy. All spaces of the threads were filled by the bone tissue, and often the bone became compacted. However, other recent studies have shown that the formation of spaces between the implant body and the bone tissue (healing chambers), which are generated by the final dimension of the osteotomy and the implant design, lead to bone formation from the blood clot that occupies these empty spaces [15,16]. The potential for bone formation in different configurations of healing chambers was studied by Marin and colleagues [17] to better understand the bone repair behavior in healing chambers of different sizes and configurations.

As previously shown, several controlled animal studies report a relationship between implant design and osseointegration. However, the literature concerning the effect of healing chambers is sparse and rare in bone with low density (rabbit tibia). The rabbit tibia is formed by a very compact cortical layer surrounding a large medullary canal, which determines an absolute low density. Thus, the aim of this animal biomechanical and histologic study was to evaluate the early host-to-implant parameters (removal torque value (RTv), bone-to-implant contact (BIC%), bone fraction occupancy inside the threads (BAFO%), and osteocyte count inside the threads (Ost)) in different implant designs in the rabbit tibia after a healing period of two months.

## 2. Experimental Section

### Materials and Methods 

**Figure 1:**
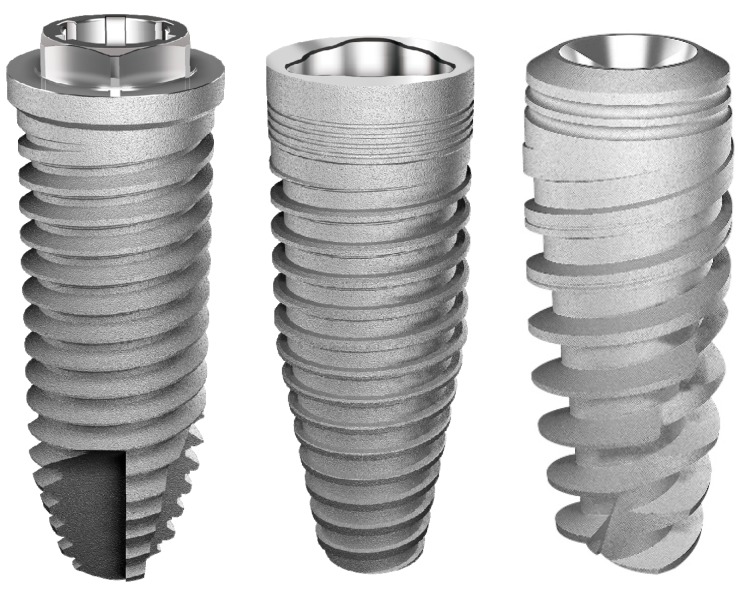
Representative image of the titanium implant macrogeometry used.

**Figure 2:**
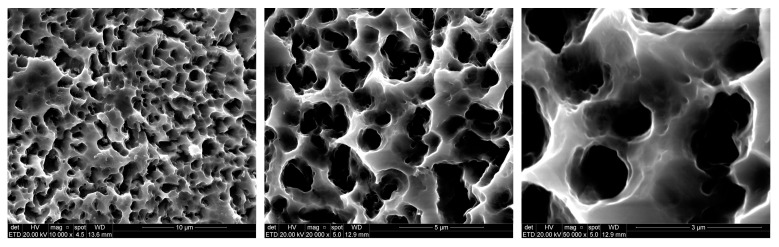
Images in different increase obtained by scanning electronic microscopy (SEM) of the surface of all sample groups.

Implant Models and Group Distribution:

Sixty titanium implants manufactured using commercially pure titanium grade IV (Derig Produtos Odontológicos Ltda, São Paulo, SP, Brazil) were used in this study. All implants used in this study were 8.5 mm in length and 3.50 mm in diameter, with a conical macrogeometry. Titanium implants with three different thread configurations were evaluated (Figure 1): Group 1 (G1) had a conventional “v” thread-shaped implant design; Group 2 (G2) had square threads; and Group 3 (G3) was the experimental group, with longer threads (healing chamber). The surface treatment of all titanium implants was performed with double-acid conditioning using hydrofluoric acid plus sulfuric acid with controlled time and temperature determined by the Company (Derig, São Paulo, Brazil), as shown in Figure 2. The surface treatment resulted in rugosity with Ra values around 0.75 µm, in accordance with the information provided by the manufacturer. All implants samples were prepared (washed, decontaminated, sterilized, and packaged) in accordance with the sanitary standards required for the commercialization of these materials.

Animal Selection and Care:

Ten white New Zealand rabbits with a height of 4.5 ± 0.5 kg were included in this randomized study. The animals received the standards care and management applied in the previous studies performed by our research group [9]. International guidelines of the animal studies were applied. The study was approved by the Animal Experimentation Committee (# 02-17UniRV), Faculty of Veterinary of University of Rio Verde (Rio Verde, Brazil). Sixty titanium implants (*n* = 20 per group) were installed in both tibias (*n* = 3 per tibia). The randomized distribution of the implants was performed using the site www.randomization.com. For the surgical procedures the animals were anesthetized through intramuscular injection of a combination of 0.35 mg/kg of ketamine (Vetanarcol, König S.A, Buenos Aires, Argentina) and 0.5 mg/kg of xylazine (Rompum^®^ Bayer S.A., São Paulo, Brazil). In both medial areas of the tibias the hairs were scraped to facilitate surgical procedures and to avoid contamination, and were cleaned with povidone–iodine solution. Then, the incision was performed with an extension of ~30 mm in length in each tibia and from 10 mm of the knee position to the distal direction. The soft tissues were separated, and the bone was exposed. The beds to insert the titanium implants were prepared using the drill sequence and speed determined by the manufactured of the implant system, under intense distilled water cooling. The implants were manually inserted with ~15 N of torque, with 10 mm between them. The first implant was installed a ~10 mm distance from the articulation, seeking to obtain a more uniform bone, and all implants were stabilized bicortically. The suture was made using a simple point with nylon 4-0 (Ethicon, Johnson & Johnson Medical, New Brunswick, NJ, USA). A single postoperative dose of 0.1 ml/kg of Benzetacil (Bayer, São Paulo, Brazil) was administered through the intramuscular (I/M) route in each animal. For the control of the pain, three I/M anti-inflammatory doses (one per day) of 3 mg/kg of ketoprofen (Ketoflex, Mundo Animal, São Paulo, Brazil) were administered. All animals were euthanized 2 months after the implantation surgeries using an overdose of anesthesia. Then, the bone blocks of both tibias were removed and the tibias of five animals were used for the torque removal test; the other five animal samples were immediately fixed by immersion in neutral formalin at 4%.

**Figure 3:**
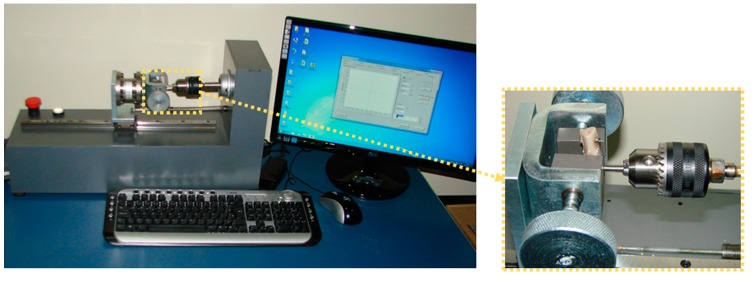
Image of the torque machine used to measure the removal torque value of the samples.

Removal Torque Analysis:

A total of 10 implants of each group were retrieved immediately after removal of the animals. Using a torque testing machine (CME, Técnica Industrial Oswaldo Filizola, São Paulo, Brazil), which is fully controlled by software DynaView Torque Standard/Pro M (Basingstoke, Hampshire, United Kingdom) (Figure 3), the measurements of the maxima force to removal the implants in reverse rotation and the mean of removal torque values were calculated for each group.

Specimen Processing and Histomorphometric Analyses:

All the samples were immediately immersed in 10% buffered formalin and maintained in this solution for 7 days. Then they were dehydrated in an ethanol solution sequence and included in a historesin (Technovit 7200 VLC, Kulzer, Wehrheim, Germany). Sections were performed using a machine IsoMet 1000 (Buehler, Lake Bluff, IL, USA). One slide was obtained for each specimen. The slides were stained using the picrosirius–hematoxylin technique for staining. Histomorphometry was carried out using a transmitted light microscope (E200, Nikon, Tokyo, Japan). For the histometry, the software ImageTool for Microsoft Windows™ (version 5.02, University of Texas Health Science Center, San Antonio, CA, USA) was used.

Percentage of BIC (BIC%) was defined as the amount of bone tissue in contact to the titanium surface. The measurements were made throughout the entire extent of the implant. The BAFO% was defined as the fraction of occupancy bone tissue within the threaded area. All threads were measured and included in the statistical analysis.

The osteocyte density was conducted at 200×, similar to other studies [18,19]; osteocyte density was obtained using the ratio of the osteocytes number, counted manually for each specimen in 10 different fields, to the bone tissue area (mm^2^), with the above-mentioned software package. The mean and standard deviation of histomorphometric variables were calculated for each implant, then for each group.

**Figure 4:**
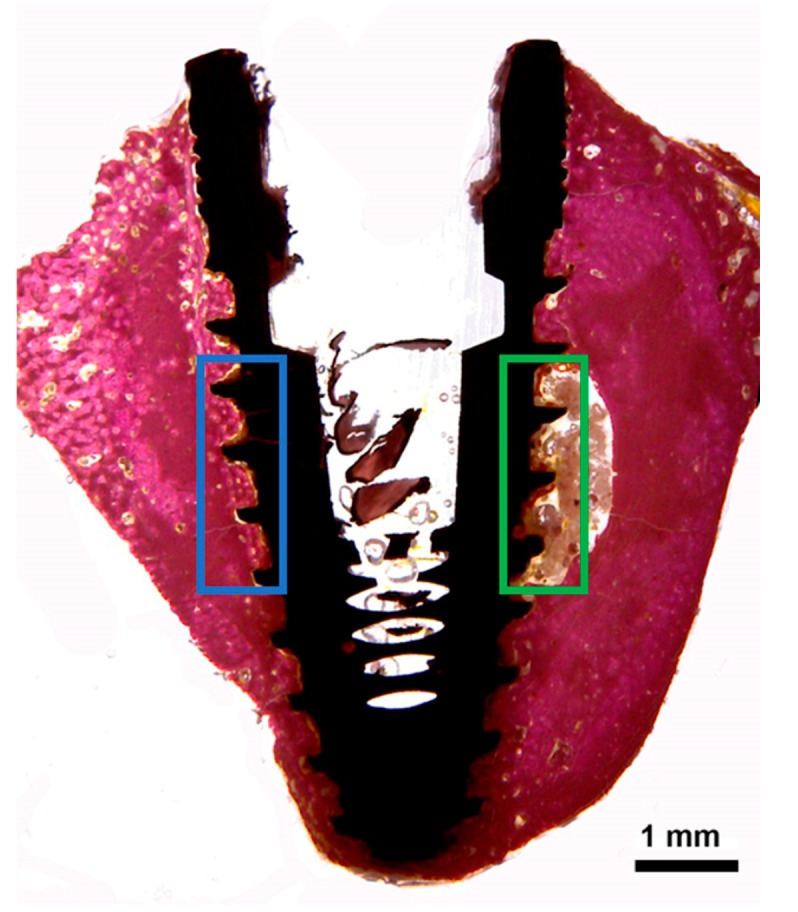
Schematic image to show the areas in the center of the implants that were evaluated by descriptive method. The blue rectangle represents side 1 (implant near the cortical bone), and the green rectangle represents side 2 (implant in contact with the medullar bone portion).

Descriptive histological observations were made in the center area of the implants, corresponding to the side where the implants stayed near the cortical bone (side 1) and the side where the implants stayed in contact with the medullar portion, as shown in the scheme of Figure 4.

Statistical Analysis:

The ANOVA one-way statistical test was used followed by Bonferoni’s multiple comparison test to determine individual difference among groups. Calculations were performed using GraphPad Prism version 5.01 for Windows (GraphPad Software, San Diego California USA, www.graphpad.com). All analyses considered the 5% level of significance.

## 3. Results

Clinical Observations:

After 2 months, all implants presented osseointegration, tested clinically at the time of retrieval, and did not present clinical evidence of inflammation or infection. Therefore, a total of 60 experimental implants (*n* = 20 implants per group) were evaluated.

Removal Torque Analysis:

The three groups presented very similar mean RTv values, without statistical differences between them (*p* > 0.05). The data are summarized in the Table 1.

**Figure 5:**
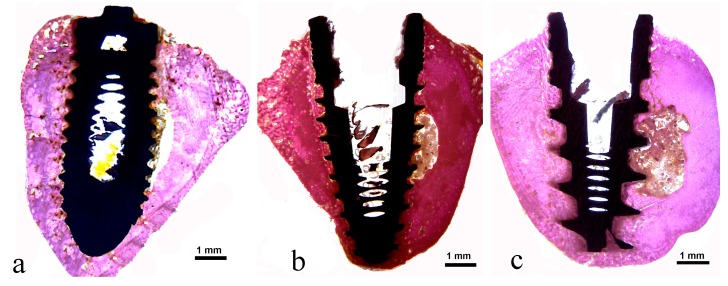
Representative histological images of samples: (**a**) Group 1, (**b**) Group 2, and (**c**) Group 3.

Histological and Histomorphometric Results:

The histologic analysis demonstrated a healthy peri-implant bone tissue surrounding all experimental implants. Neither epithelial downgrowth nor inflammatory cell infiltrate were observed in all evaluated groups. In a closer view, the interface between implants and bone tissue was filled by new bone at different levels. A newly formed peri-implant bone was observed in close contact with the implant surface, especially in the coronal area. In some portions of the bone–implant interface, in the coronal and apical portions of the implants, osteoblasts were depositing osteoid matrix directly onto the titanium implant surface of all groups (Figure 5).

**Figure 6:**
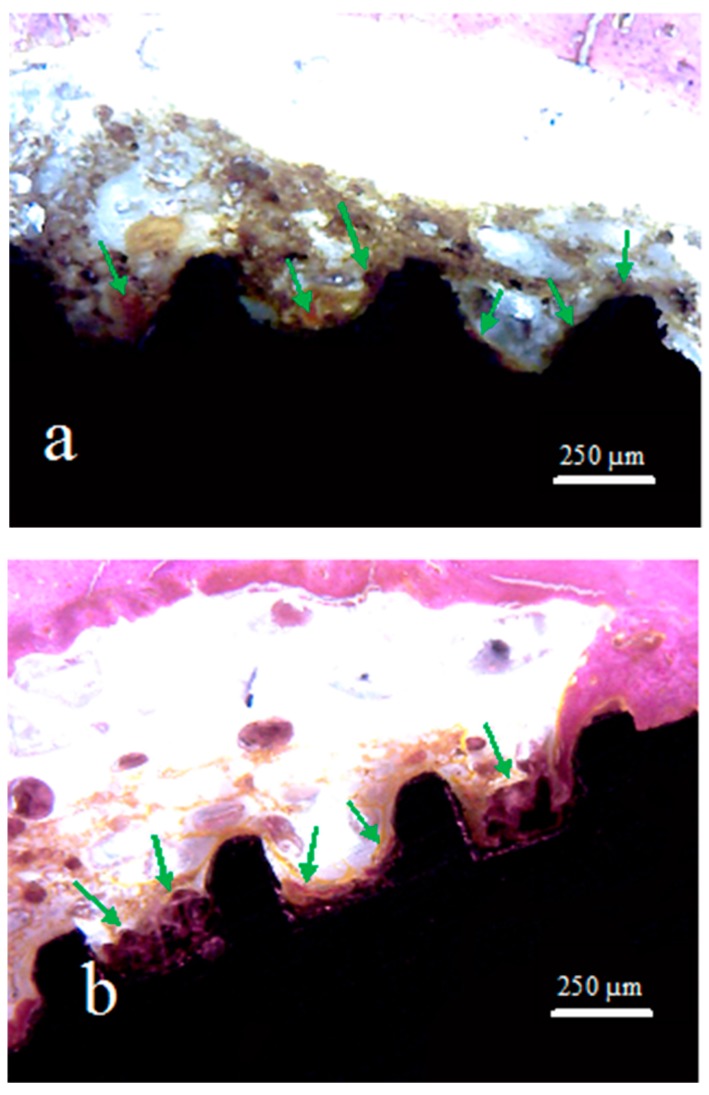

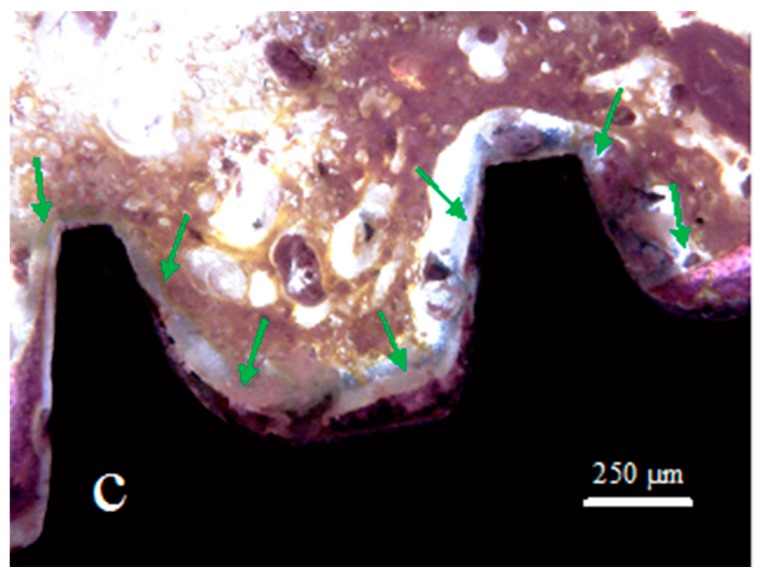
Representative histological images of samples: (**a**) Group 1, (**b**) Group 2, and (**c**) Group 3. The green arrows indicate the areas with new bone formation inside of the implant threads.

In the central portion of the implants corresponding to side 2 (bone marrow tissue), as shown in the scheme of Figure 4, different levels of bone formation were observed in the three groups. A thin layer of newly formed bone tissue was interposed on the surface of the implant in this area in G2 and G3, while in G1 a few signs of neoformation were found (Figure 6).

**Figure 7:**
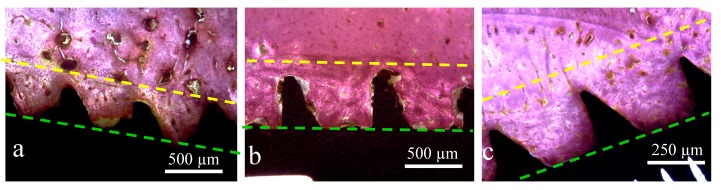
Representative histological images of samples: (**a**) Group 1, (**b**) Group 2, and (**c**) Group 3. The space between the lines showed the different amount of bone reaction (stimulation) from the implant body (green line) to the native bone tissue (yellow line) of each group.

In the side that was in contact with the portion of the cortical bone (side 1), the implants of all groups showed a different behavior of the bone-to-implant contact (Figure 7). The histological characteristics observed in the bone tissue showed that the amount of bone reaction and/or stimulation from the body of the implant to the native bone presents a signal of proportionality of the size (depth) presented by the implant threads and the extension of these events.

**Figure 8:**
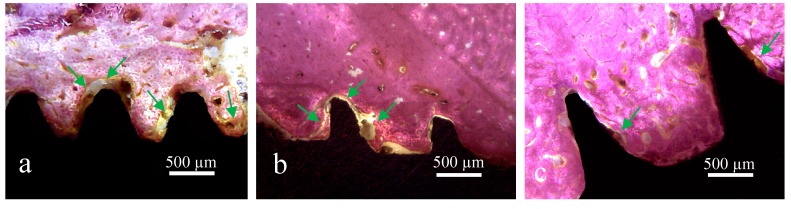
Representative histological images of samples: (**a**) Group 1, (**b**) Group 2, and (**c**) Group 3. The green arrows indicate the areas with collagen tissue around of the implant surface.

Detailed observation of the bone in proximity to the V threads (G1) and squared threads (G2) revealed bone tissue with more collagen areas compared to bone inside the healing chambers of the G3 implants (Figure 8).

**Figure 9:**
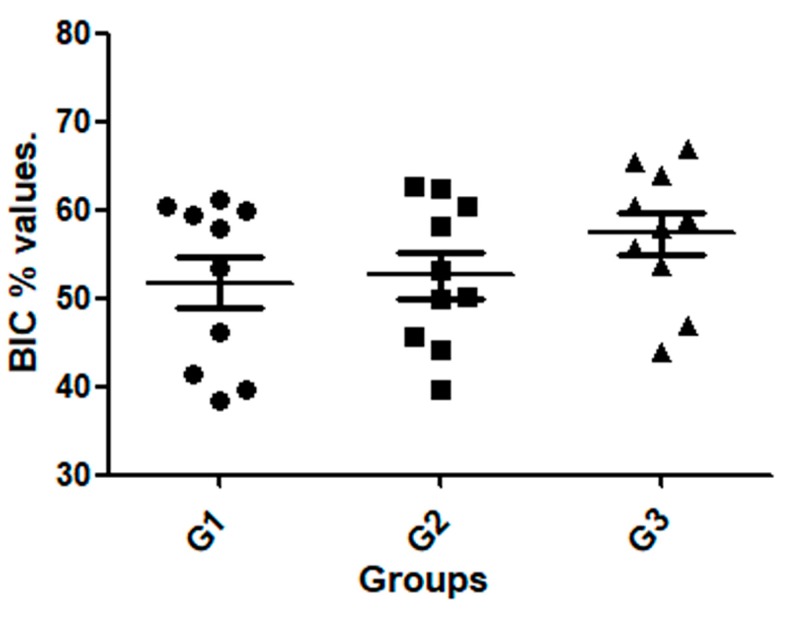
Graph showed the value distribuition in each group.

In general, the newly formed bone surrounding around all implants showed early stages of remodeling. Although there were no significant differences in the BIC% among the three groups (*p* = 0.2935), a higher tendency for BIC% median was observed for the G3 implant group. BIC% values for G1 ranged between 38.5 and 60.2%, while for G2 these values ranged between 39.6 and 62.7%, and for the G3 thread the values ranged from 44.0 to 66.8%. The data of measured values are presented in Table 2 and the distribution shown in the graph attached of Figure 9. The statistical test analysis between groups are presented in Table 3.

**Figure 10:**
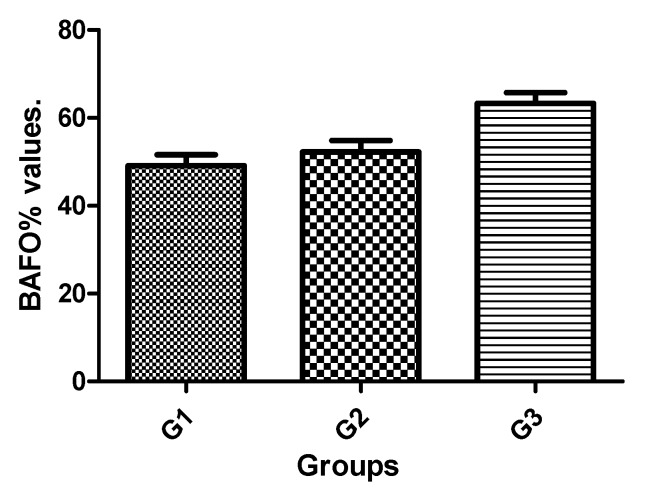
Bar graph of the BAFO% mean and standard deviation.

One-way ANOVA showed significant changes, with a higher bone fraction occupancy (BAFO%) for G3. The mean BAFO% observed for G1 was 49.07 ± 8.18%, while for G2 it was 52.21 ± 8.34%, and for G3 it was 63.28 ± 7.94%. The statistical differences between groups are presented in Table 4 and the bar graph of Figure 10 shows the data for visual comparison between the groups.

**Figure 11:**
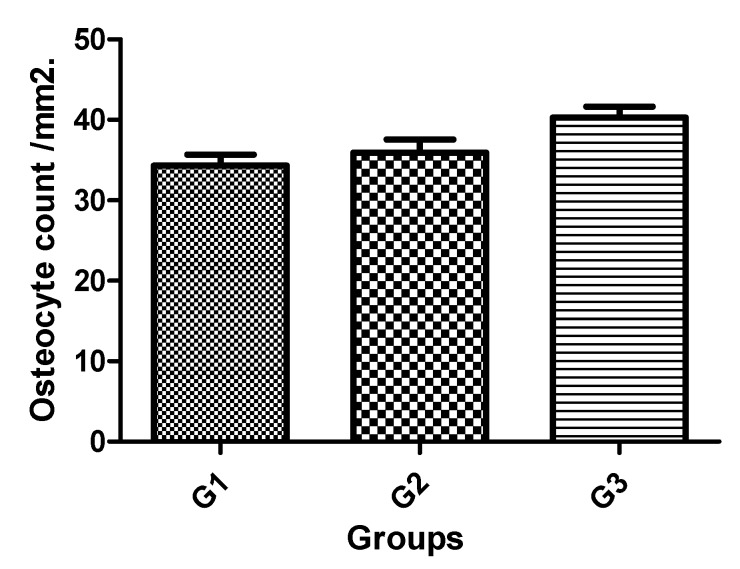
Bar graph of the osteocyte count per mm^2^ mean and standard deviation.

The osteocyte count at distance and near to implant surface was also measured. Although a tendency to display higher mean value was observed for G3 implants compared with G2 and G1, this difference was statistically significant between Group 3 and Group 1. The Ost count mean value adjacent for G1 was 34.31 ± 4.37 /mm^2^, for G2 it was 35.94 ± 5.09 /mm^2^, and for G3 it was 40.28 ± 4.36/mm^2^. The statistical differences between groups are presented in Table 5, and the bar graph of Figure 11 shows the data to visual comparison between the groups.

## 4. Discussion

This study demonstrated increased bone density values to implants with healing chambers compared to implants with squared and conventional thread design. Recently, several studies have shown that macrogeometry of the implant could influence early bone healing at the tissue/implant, interface increasing bone formation [15,16,17,18,20]. However, our results showed that these processes did not influence the bone-to-implant contact, at least at two months follow-up. The authors speculated that healing chambers influenced the bone tissue response at the new bone tissue formation into the threads and not at the bone interface. The healing chamber design has a particular blood clot apposition during both implant placement and bone healing, as previously demonstrated in pre-clinical studies [15,17]. In this sense, the newly formed bone filled a large portion of the thread chambers in G3, presenting higher values of bone fraction occupancy and presence of osteocytes in comparison with the other two groups. Complementary, some histological sections showed osteoblasts lining the newly formed bone, although this feature was less evident in the other groups.

Removal torque measurement is a frequent method of in vivo biomechanical analysis used to evaluate the interaction force of the bone and implant contact [21,22]. The data obtained in the reverse torque test of implants that are osseointegrated can indicate the levels of contact between bone and the surface of the implant, as well as the quality of this new bone formed (degree of mineralization) after its healing [23]. In the present study, three implants groups had different thread configurations after implantation; the test results showed similar RTv between the groups and it is concluded that there is no significant effect between groups on the maturation of bone tissue around the implants, with non-significance set at *p* > 0.05. High removal torque values were obtained in the implants in all groups, which probably is related surface treatment characteristics presented by the implants used in these studies, as this variable (surface roughness) is directly related to the values obtained in this type of analysis (RTv test) [24]. Ivanoff et al. (1996) [25] reported that removal torque was closely related to the bone–implant contact and the amount of bone inside the threads. In the present study, a special apparatus that allows for computer-controlled torque was used, which decreased the possibility of introducing operator error.

The healing chamber of G3 presented higher amount of BAFO%, indicating that the cellular reaction differed between the implant thread configurations. Previous studies in animal models have shown that longer threads (healing chambers) inserted in the cortical bone did not increase the BIC%, but increased implant biomechanical fixation at early times when compared to the conventional thread design [26]. This occurrence may be explained based on bone quality and quantity. The cortical bone offers a more organized vital structure when compared with the type IV bone present in the trabecular portion. Our results depicted higher BIC% for all groups in areas even of low bone density (rabbit tibia), suggesting that the implant surface topography played a pivotal role in early host-to-implant interaction in bone presenting low-density levels, as recently suggested by Soto-Peñaloza et. al. [27]. However, the abundant presence of osteogenic tissue throughout the chamber area and closer interaction with the implant surface observed for the two-month period possibly resulted in the significantly higher degrees of BAFO%, ratifying a previous animal study that showed that surface wettability is beneficial in hastening osseointegration in healing chambers at early periods [28].

Osteogenesis at the bone-to-implant interface is influenced by several biological and physical mechanisms. In turn, each of these events is affected by physicochemical interaction between the molecules and cells in the implant environment [29]. The implant surface topography characteristics, as well as the specific properties of individual proteins, determine the organization of the adsorbed protein layer. Earlier studies on dental implant surface topography and chemistry have shown that the implant surface topography itself can affect both the osteoblast gene expression and cell differentiation [30]. In addition, the results of the present study have shown a higher mean without significant difference for osteocyte density at bone regions in close proximity with the implant. Although the role of the osteocytes is not totally clear, an important role in the regulation of bone skeleton remodeling has been shown [16]. Modifications in the osteocyte environment release growth factors and cytokines that affect osteoblasts and osteoclasts. Woven bone has been found to have a greater number of osteocytes than lamellar bone [31]. Osteocyte density has been reported to be inversely proportional to bony mass and the osteocytes seem to be involved in the maintenance of the functional bone matrix [19]. Consequently, it may be suggested that the healing chamber configuration as presented in G3 could influence also osteocyte index. Still, G3 depicted a tendency to have a higher density of osteocytes, with a statistical difference among the groups, showing higher quantities of new bone rates inside of the threads areas. Further characterization and correlation between the osteocyte index and other histomorphometric parameters must be done to clarify this process.

## 5. Conclusions

Within the limitations of this animal study, with regard to the biomechanical and histometric analysis the histological data obtained in rabbit tibia confirmed that the healing chamber design could positively influence/modulate early bone tissue response after the two-month healing period evaluated.

## Figures and Tables

**Table 1:** Mean, standard deviation (SD) and median of removal torque values measured for each group in Newton per centimetre (Ncm). G: group.

Parameter	G1	G2	G3
Mean ± SD	73.9 ± 3.51	71.5 ± 4.33	73.3 ± 4.15
Median (range)	73.5 (60–81)	72.0 (63–80)	73.4 (59–80)

**Table 2:** Table data (mean, standard deviation, and median) and graph values distribution of the bone-to-implant contact percentage (BIC%) measured around of the surface of each sample.

Group	BIC% ± SD	Median
G1	51.8 ± 9.39	55.7
G2	52.6 ± 8.12	51.6
G3	57.4 ± 7.58	58.5

**Table 3:** Bonferroni’s multiple comparison test to compare the BIC% values between the groups.

Group Comparison	Mean of Difference	*t*	Significant *p* < 0.05	95% Confidence Interval of Difference
**G1 vs. G2**	−0.8400	0.2237	No	−10.43 to 8.747
**G1 vs. G3**	−5.580	1.486	No	−15.17 to 4.007
**G2 vs. G3**	−4.740	1.262	No	−14.33 to 4.847

**Table 4:** Bonferroni’s multiple comparison test to compare the bone fraction occupancy inside the threads (BAFO%) values between the groups.

Group Comparison	Mean of Difference	*t*	Significant *p* < 0.05	95% Confidence Interval of Difference
**G1 vs. G2**	−3.140	0.8608	No	−12.45 to 6.170
**G1 vs. G3**	−14.21	3.896	Yes	−23.52 to −4.900
**G2 vs. G3**	−11.07	3.035	Yes	−20.38 to −1.760

**Table 5:** Bonferroni’s multiple comparison test to compare the osteocyte count values between the groups.

Group Comparison	Mean of Difference	*t*	Significant *p* < 0.05	95% Confidence Interval of Difference
**G1 vs. G2**	−1.630	0.7889	No	−6.904 to 3.644
**G1 vs. G3**	−5.970	2.889	Yes	−11.24 to −0.6963
**G2 vs. G3**	−4.340	2.101	No	−9.614 to 0.9337
